# Antioxidants and Sports Performance

**DOI:** 10.3390/nu15102371

**Published:** 2023-05-18

**Authors:** Vicente Javier Clemente-Suárez, Álvaro Bustamante-Sanchez, Juan Mielgo-Ayuso, Ismael Martínez-Guardado, Alexandra Martín-Rodríguez, José Francisco Tornero-Aguilera

**Affiliations:** 1 Faculty of Sports Sciences, Universidad Europea de Madrid, Tajo Street, s/n, 28670 Madrid, Spain; 2 Department of Health Sciences, Faculty of Health Sciences, University of Burgos, 09001 Burgos, Spain; 3 BRABE Group, Department of Psychology, Faculty of Life and Natural Sciences, University of Nebrija, C/del Hostal, 28248 Madrid, Spain

**Keywords:** inflammation, sports performance, antioxidant, reactive oxygen species, training, microbiota, nutrition, physical activity

## Abstract

The role of reactive oxygen species and antioxidant response in training adaptations and sports performance has been a large issue investigated in the last few years. The present review aims to analyze the role of reactive oxygen species and antioxidant response in sports performance. For this aim, the production of reactive oxygen species in physical activities, the effect of reactive oxygen species on sports performance, the relationship between reactive oxygen species and training adaptations, inflammation, and the microbiota, the effect of antioxidants on recovery and sports performance, and strategies to use antioxidants supplementations will be discussed. Finally, practical applications derived from this information are discussed. The reactive oxygen species (ROS) production during physical activity greatly influences sports performance. This review concludes that ROS play a critical role in the processes of training adaptation induced by resistance training through a reduction in inflammatory mediators and oxidative stress, as well as appropriate molecular signaling. Additionally, it has been established that micronutrients play an important role in counteracting free radicals, such as reactive oxygen species, which cause oxidative stress, and the effects of antioxidants on recovery, sports performance, and strategies for using antioxidant supplements, such as vitamin C, vitamin E, resveratrol, coenzyme Q10, selenium, and curcumin to enhance physical and mental well-being.

## 1. Introduction

A substance that can slow down the oxidation of proteins, carbohydrates, lipids, and DNA at low concentrations is called an antioxidant. There are three main categories of antioxidants [1]: the first line of defense includes substances such as superoxide dismutase, catalase, glutathione reductase, and minerals such as selenium, copper, and zinc. The second line of defense includes substances such as glutathione, vitamin C, albumin, vitamin E, carotenoids, and flavonoids. The third line of defense includes a complex group of enzymes that repair damaged DNA, proteins, oxidized lipids, and peroxides. Examples include lipase, protease, DNA repair enzymes, transferases, and methionine sulphoxide reductase.An imbalance between reactive oxygen species (ROS) and antioxidant defenses results in oxidative stress, which can disrupt cellular functions and lead to various pathological conditions such as AIDS, aging, arthritis, asthma, autoimmune diseases, carcinogenesis, cardiovascular dysfunction, cataracts, diabetes, and neurodegenerative diseases such as Alzheimer’s disease and Parkinson’s dementia, among others [2].

In relation to exercise, during exercise, the metabolism speeds up due to the increased need for oxygen. This causes the release of highly reactive oxygen species (ROS) from the mitochondria. Physical exertion itself also triggers the activation of enzymes, which in turn increases the number of ROS [3], which can harm cells and contribute to muscle damage, fatigue, and immune problems. However, they also have positive effects, such as aiding in glycogen resynthesis, reducing the risk of infection, and enhancing athletic performance through adaptive responses to training. Whether ROS are harmful or helpful depends on factors such as exercise duration, intensity, fitness level, and nutrition [4].

Thus, intense exercise can increase the production of free radicals or reactive oxygen species (ROS) and nitrogen species (RONS), which can hinder muscular contractile function and cause muscle fatigue and reduced performance [5]. To combat muscle damage and fatigue and enhance performance, athletes often consume antioxidant supplements. However, recent research suggests that the positive effects of antioxidants on health and sports performance may also produce harmful effects in high doses [6]. Indeed, the American College of Sports Medicine suggests that around half of athletes use vitamin supplements to maintain fitness and boost stamina [7]; yet, from this percentage, another 50% of elite endurance and male collegiate athletes consumed doses of antioxidant supplements daily that were higher than the recommended daily allowance (RDA) [6]. This consumption and such high doses are often based on the mistaken assumption that antioxidants are unlikely to be toxic and can prevent the harmful effects of reactive oxygen species (ROS) on cell structure and function. However, there is evidence that ROS play important roles in physiological signaling pathways that regulate the response to exercise, and oxidative stress induced by physical activity is essential for training adaptation. This means that the appropriate balance between antioxidants and free radicals is necessary for obtaining physiological adaptation, and the use of antioxidants in athletes may negate the beneficial effects of ROS in normal cell signaling [8].

In this line, reactive oxygen species generated during exercise are believed to stimulate molecular pathways, including PGC1-α and MAPK, which are critical to aerobic capacity and muscle hypertrophy [9]. Some authors suggest that a transient increase in ROS induced by exercise can have positive effects, such as regulating muscle contractile activity, stimulating muscle regeneration, and improving vasodilation during exercise. However, high levels of ROS and oxidative stress can cause inflammation and damage to cells and tissues. Despite their high training demands, many endurance athletes have diets that lack sufficient antioxidants to support their physical activity demands [10]. While it is often recommended that endurance athletes take antioxidant supplements, given the many proven health benefits of exercise, this approach may not be necessary.

In relation to sports performance, some of the most commonly used anti-redox supplements by athletes with the aim of improving performance may have a potentially negative effect on physical performance, depending on the dosage. This may be the case with vitamin C, as ROS may facilitate beneficial adaptations from training, but vitamin C can hinder these adaptations. Out of 12 studies, 4 showed substantial impairments in sports performance from vitamin C doses of 1 g or more daily, potentially by decreasing mitochondrial biogenesis. Another four studies showed impairments but not significant enough to be statistically relevant. Three out of four studies reported performance improvements but only when vitamin C was taken acutely or for a short period of up to 1 week. The conclusion drawn in this review was that consuming lower doses of vitamin C from fruits and vegetables (up to 250 mg daily) can reduce oxidative stress and offer health benefits without affecting training adaptations negatively [11,12].

Despite the potential negative effects of vitamin C on performance, previous studies have proposed ways in which other antioxidants could enhance either muscular or cardiovascular performance. In this line, vitamin E has been suggested by researchers as a helpful supplement to maintain the structure of red blood cells during exercise at high altitudes, which could be translated into training in hypoxia [13]. Furthermore, researchers also suggest that polyphenols, including quercetin, may induce mitochondrial adaptation and improve peripheral circulation, similar to exercise [14]. In this line, high doses of certain polyphenols, such as quercetin and resveratrol, may also promote mitochondrial biogenesis by activating peroxisome-proliferator-activated receptor gamma coactivator 1-alpha through adenosine-monophosphate-activated protein kinase, leading to improved endurance capacity [15], also increasing nitric oxide synthesis in the endothelium, improving blood flow.

Beetroot juice, which is rich in polyphenols and inorganic nitrate, can also impact oxygen utilization and blood flow [16]. Spirulina contains various antioxidants, such as tocopherol, β-carotene, polyphenols, and phytocyanins, all of which can reduce exercise-induced reactive oxygen species [17]. Another example would be N-acetyl cysteine (NAC), which can directly scavenge reactive species and provide cysteine for glutathione synthesis, an essential intracellular antioxidant [18].

In summary, physical exercise is a fundamental tool for health and physical performance. However, when the training load or volume is too high, ROS appear as a consequence. In this line, athletes seek ways to mitigate the negative effects of ROS at a physiological level; however, the lack of knowledge and scientific literature may lead the athletes to an inappropriate dose and intake of antioxidants, thus having a negative effect by inhibiting molecular signaling pathways in processes such as angiogenesis and muscle hypertrophy. ROS and RONS are essential in the processes of adaptation to training, through an attenuation of inflammatory factors and oxidative stress as well as correct molecular signaling; therefore, a balance is necessary.

Thus, the present narrative review provides a comprehensive overview of the complex interplay between ROS, antioxidants, and exercise and sheds light on the potential benefits and limitations of antioxidant interventions in the context of sports performance. The aim is to explore the effects of ROS production on sports performance, training adaptation, and recovery, as well as the role of antioxidants in mitigating the harmful effects of ROS [18,19,20]. Understanding performance from a holistic perspective, such as microbiology and the relationship between antioxidant capacity and the microbiota, shows the potential benefits of an antioxidant-rich diet and antioxidant supplementation for sports performance. Finally, practical applications and strategies for antioxidant supplementation in sports competitions, including the timing and dosage of antioxidant supplements, will be studied.

## 2. Materials and Methods

A literature search was conducted for this study using both primary and secondary sources, including scientific articles, bibliographic indexes, and databases such as PubMed, Scopus, Embase, Science Direct, Sports Discuss, ResearchGate, and the Web of Science. MeSH-compliant keywords such as antioxidants, exercise, performance, adaptation, recovery, supplementation, and the gut microbiota were used to search for articles published from 1 January 2003 to 1 January 2023. To determine the inclusion criteria, a team of six review authors screened the titles and abstracts of all retrieved manuscripts. Exclusion criteria were then applied to studies that used old data outside the proposed timeline, had inappropriate topics that were not relevant to the study’s focused purpose, or were not written in English. The same team of nine review authors who conducted the study selection extracted the information from the selected studies. The studies were selected independently, and the results were discussed to produce the present narrative review.

## 3. Physical Exercise and Reactive Oxygen Species Production

**Figure 1:**
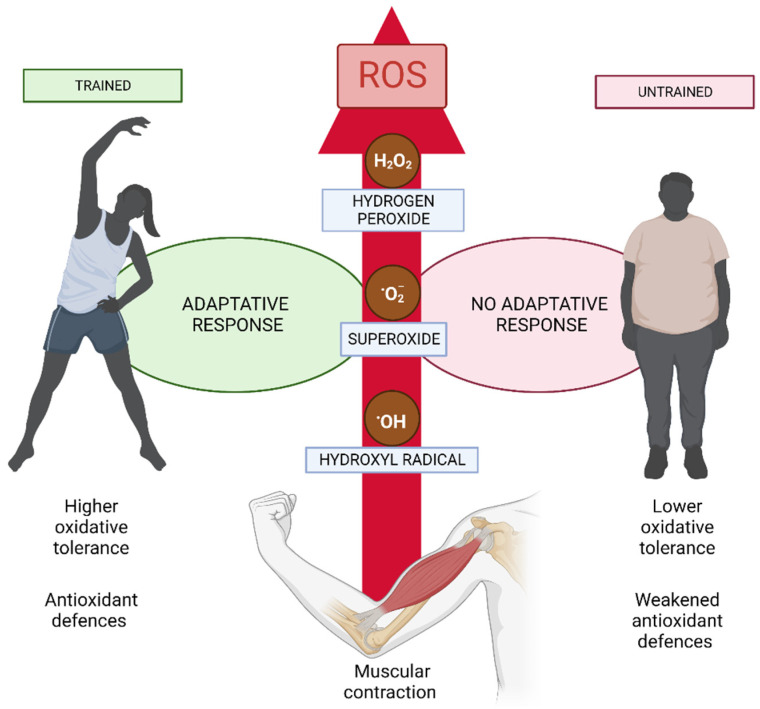
Response against ROS in trained and untrained individuals.

Frequent physical activity provides systemic benefits, including the enhancement of brain function. However, unaccustomed strenuous exercise can lead to muscle injury, inflammation, and oxidative stress. Reactive oxygen species (ROS) are produced during repeated muscular contraction, with mitochondria, NADPH oxidase enzymes (NOX/DUOX), and xanthine oxidase (XO) being the main sources of ROS during exercise (Figure 1). While mitochondria and XO produce oxygen as by-products of oxidative phosphorylation and purine metabolism, respectively, NOX enzymes are responsible for producing superoxide (O•^−2^), hydrogen peroxide (H_2_O_2_), and hydroxyl radical (•OH) [21] (Figure 1).

Normal muscular force requires moderate levels of ROS, but excessive ROS can lead to muscle exhaustion and contractile failure. However, some studies have shown that oxidants at low concentrations may be crucial for enhancing muscle contraction, as exogenous antioxidant therapies have been shown to decrease muscular contractility, but this effect is reversed when hydrogen peroxide is added (Figure 1). On the other hand, excessive ROS production combined with weakened antioxidant defenses due to strenuous exercise or other stresses may result in oxidative stress and tissue damage [22]. As a result, the adaptive response to regular exercise is characterized by the upregulation of the enzymatic antioxidant system and the control of oxidative damage (Figure 1).

Under physiological circumstances, it is well established that mitochondrial cellular respiration results in the production of ROS. Exercise-induced ROS formation can occur through cytoplasmic glucose metabolism and NADH regeneration, as well as increased ATP hydrolysis rates and purine metabolism, which may be associated with the increased energy metabolism demand after physical activity. Fast-twitch fibers, which have high levels of glycogen and use glycolysis as their primary ATP source, are the main sources of lactate release, and lactate is generated in an intensity-dependent manner. Data suggest that NADPH oxidases could generate ROS from the elevated intramuscular NADH levels resulting from energy metabolism. Recent research has shown that the ability of intestinal cells to convert lactate into pyruvate results in the production of NADH, which can then activate NADPH oxidases. In this regard, the NOX4 enzyme may function as a metabolic sensor by connecting exercise-induced ROS generation to ATP metabolism due to the presence of an ATP-binding motif in its structure and its control by ATP [23]. Specifically, NOX and XO are the major endogenous ROS producers in skeletal muscle. While superoxide produced by mitochondria may be detected in both resting and active muscles, evidence has suggested that mitochondria may not be the primary generator of ROS in exercising muscles. On the contrary, NOX is a significant ROS producer during muscle contractions, contributing more to cytosolic superoxide than mitochondria. During exercise, NOX-induced ROS in T-tubules can dramatically affect ryanodine receptor type 1 to increase calcium (Ca^2+^) release and muscular contractions [24]. Moreover, XO promotes the formation of extracellular superoxide during isometric contraction by being present in the endothelium and cytoplasm of the muscle. This XO-derived superoxide is essential for the production of muscular power [25]. Considering the quantity of exercise performed, untrained subjects are more susceptible to the negative consequences of increased oxidative stress, whereas trained individuals often have diminished effects due to higher oxidative tolerance (Figure 1). According to an analysis of the cell signaling pathways controlling these skeletal muscle changes, a recent systematic review stated that redox signaling pathways play a critical role in the regulation of muscle remodeling during both exercise and extended periods of rest [26,27,28].

Normally, intracellular antioxidants such as bilirubin, vitamin E, or vitamin C are located within cells, the cytoplasm, and organelles (such as mitochondria) to protect muscle fibers from damage induced by reactive oxygen species (ROS). However, increased generation of ROS during vigorous and exhaustive exercise can counteract these protective processes [29,30,31,32]. Indeed, although most studies have focused on the harmful effects of exercise-induced oxidative stress on muscles, researchers have recently reported the importance of ROS in initiating and regulating the body’s adaptive responses to exercise [33,34]. Significant routes in the adaptive responses to training have been proposed. The activation of the primary signaling pathways involved in muscle adaptation is thought to depend on the generation of mitochondrial reactive oxygen species during routine exercise [35]. The main regulator of antioxidants as well as other cytoprotective cofactors is nuclear factor erythroid 2-related factor (Nrf2), a redox-sensing transcription factor that is responsible for the increased antioxidant defense mechanism [36]. Enhancing mitochondrial biogenesis through elevated peroxisome proliferator-activated receptor-gamma coactivator-1 alpha (PGC-1 alpha) gene expression is another adaptation to exercise. The redox-sensitive upstream signals that regulate PGC-1 alpha expression include mitogen-activated protein kinase (MAPK) and nuclear factor (NF)-kB [37]. Other cellular signaling pathways, such as 5-adenosine monophosphate-activated protein kinase (AMPK), p38 mitogen-activated protein kinases (p38 MAPK), and Ca^2+^/calmodulin-dependent protein kinase, are also triggered by muscle contraction and increased PGC-1 alpha mRNA levels (CaMK) [38]. Importantly, failure to trigger some of the aforementioned exercise effectors may result in a disruption in redox signaling caused by antioxidant supplementation. For instance, PGC-1 alpha was shown to be triggered in electrically stimulated myotubes by cAMP response element binding protein (CREB) signaling via superoxide produced by NOX2 [39]. Previously, PGC-1 alpha was believed to be the main player in adaptations to endurance exercise. However, resistance training was found to induce PGC-1 alpha 4, a shortened splice version of PGC-1 alpha, but not endurance training. The expression of PGC-1 alpha 4 was linked to the hypertrophic muscle response but was unable to enhance IGF-1 or the same group of oxidative genes, such as TFAM, PGC-1 alpha, and NRF2 [40]. Therefore, recent research has identified PGC-1 alpha as a key factor in coordinating both resistance (myogenic) and endurance (oxidative) adaptations to exercise.

In this line, endurance exercise increases metabolism, which often leads to induced oxidative stress and injuries. It is estimated that aerobic exercise causes a 1–3 times increase in O•^−2^ during muscle contraction. However, mitochondria only contribute a minor amount to the production of O•^−2^ during aerobic exercise [22,25]. Sprinting, on the other hand, primarily uses anaerobic energy pathways due to its high energy demands. Only a small amount (0.15%) of O•^−2^ is generated in the mitochondria during sprinting. The relatively reduced oxygen consumption and higher ADP (state 3) during sprints result in lower ROS generation in skeletal muscle mitochondria than typical [41]. NOX is one of the potential locations for O•^−2^ generation during rapid muscle contraction. Another significant factor for the generation of ROS is the activation of XO caused by an increase in hypoxanthine during and after sprints [42]. It is critical to emphasize that only a small number of studies have attempted to examine ROS-mediated cellular signaling after resistance training programs. For instance, greater research is required to determine whether PI3/Akt functions as a redox-sensitive cascade to promote hypertrophy [43]. Although physical activity associated with sports performance will be covered in detail in the following sections, it is necessary to mention that the formation of ROS during skeletal muscle contraction and physical activity has been the subject of intense discussion for the past four decades and still is. Reactive oxygen species (ROS) are still difficult to detect due to their instability and methodological challenges, but significant advancements have been made recently, particularly in the development of imaging techniques and the use of redox-active biosensor compounds to detect ROS produced in subcellular environments.

## 4. The Effect of Reactive Oxygen Species on Sports Performance

**Figure 2:**
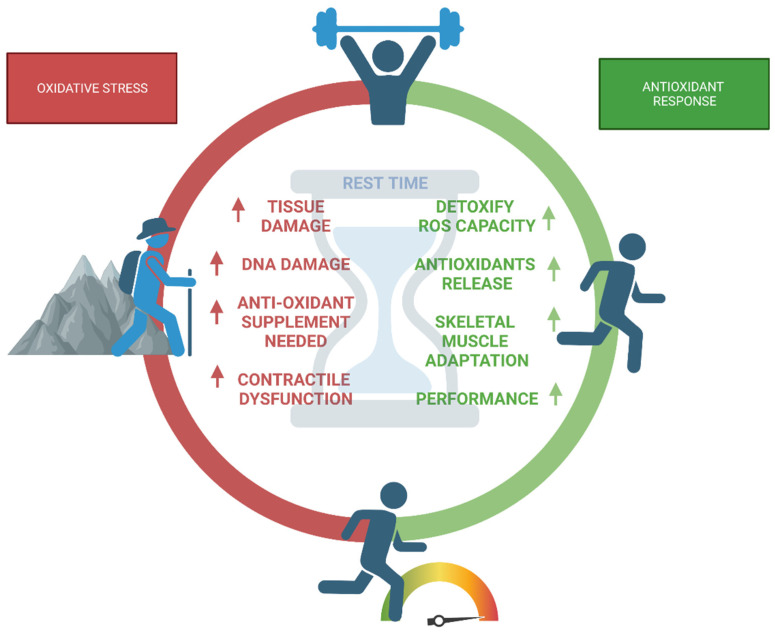
ROS and antioxidant response in sports performance.

The precise redox pathways causing exercise-induced oxidative stress and exercise-induced adaptability are still not fully understood. Our comprehension of muscle exhaustion and recovery after exercise, as well as the creation of prospective methods for ROS measurement in active muscles, may be improved by investigating ROS pathways. Although there is growing evidence linking exercise to an increase in oxidative stress, systematic research on how exercise-related activities (such as exercise type, intensity, and duration) affect ROS generation is still lacking [21]. Traditionally, exercise is separated into aerobic and resistance training components. Aerobic exercise strengthens the cardiovascular system and decreases reactive oxygen species and several ROS-related responses dramatically as exposed in Figure 2. As a direct consequence, it is critical to distinguish between training methods that call for a high level of athletic performance. The knowledge presented here is focused on endurance, strength, and high-intensity training, as well as mountain sports because of the distinctive properties of ROS at high altitudes (Figure 2).

Exercise-induced oxidative stress is mitigated by aerobic exercise, which boosts antioxidant levels and antioxidant enzyme expression [44]. Conversely, resistance training promotes structural (i.e., muscle fiber) and neurological adaptations while also causing an increase in oxidative and inflammatory stress. Due to elevated levels of oxidative and inflammatory stresses brought on by resistance training, the recovery time reduces oxidative stress and allows for the repair of minor injuries. Indeed, in skeletal muscle, intense physical activity can generate high production of ROS and reactive nitrogen species (RNS). A large concentration of reactive species might be hazardous to the organism if it is not neutralized by the available antioxidant enzymes, depending on the activity level, duration, frequency, sex, age, and fitness [45]. For instance, locally generated IGF-1 made by muscle cells, which acts autocrine and paracrine, is thought to be the most important hypertrophic stimulation. Among the compounds produced by IGF-1 posttranscriptional regulation is mechano growth factor (MGF). Both proteins are regarded as crucial mediators of exercise-induced skeletal muscle growth, which can occur via ROS signaling. During exercise-mediated hypertrophy signaling, phosphatase and tensin homolog (PTEN) and protein phosphatase 2A (PP2A) regulate two essential steps in PI3K/Akt signaling and are susceptible to H2O2-mediated signaling. In addition to the temporary suppression of PTEN, H2O2-mediated signaling causes the phosphorylation of PTEN, leading to its destruction. Furthermore, isometric exercise is widespread in daily activities involving static positions, such as carrying heavy objects. Many biomarkers of oxidative stress have been investigated in response to isometric exercise, but the results have been inconsistent. Isometric contractions, for instance, result in elevated levels of hydroperoxide and blood protein carbonyls. Nevertheless, malondialdehyde levels in plasma remain unchanged [21,34]. Additionally, a small number of studies have also evaluated the oxidative stress caused by eccentric exercise, a physical activity that can cause sarcolemma inflammation and consequent ROS overproduction and muscle injury [46]. However, over time, the adaptations brought on by consistent, vigorous exercise diminish the overall oxidative stress [47].

Focusing on high-intensity exercise, a recent study assessing a variety of these activities in athletes deduced that it mainly results in the production of extra reactive oxygen species (ROS) in skeletal muscle. Therefore, athletes must preserve increased ROS scavenging activity in the body [48]. The active sympathetic nervous system can contribute to the generation of ROS in response to vigorous exercise. As a result, Bors et al. showed that administering adrenaline significantly raised H2O2 levels in vitro [49]. According to earlier studies, the body’s oxygen intake during intense activity is 10–15 times larger than at rest, and the oxygen uptake by active muscles is approximately 100 times greater [50]. Although it has been hypothesized that an increase in ROS and free radical production in the body may result in tissue damage, inflammation, and various oxidative diseases, extreme anaerobic exercise significantly boosted the scavenging activities of various serum reactive oxygen species and free radicals, according to recent findings [48]. In this regard, the alterations in these scavenging activities vary significantly for each reactive oxygen species and free radical in response to intense exercise stress. When Sawada et al. compared medium–high power groups with medium–low power groups, the rate of increase in OH was substantially larger in the medium-high power group compared to the medium-low power group. This suggests that the amount of antioxidant myokines released immediately following exercise varies between individuals. In other words, exercise performance may be connected with an athlete’s ability to release antioxidant myokines. The findings show that those with greater exercise performance are more susceptible to the oxidative stress induced by exercise interventions, suggesting that those who are able to respond to the rapid oxidative stress caused by high-intensity exercise are capable of achieving greater performance (Figure 2) [48,51]. However, there is evidence that NOX2 is activated by high-intensity interval training and that functional NOX2 is necessary for long-term training adaptation. Examples of this data include enhanced muscle protein expression of antioxidant defense enzymes, mitochondrial enzymes, and hexokinase II, as well as increased mitochondrial network volume and decreased mitochondrial fragmentation [52]. In addition, XO activation induced by an increase in hypoxanthine during and after sprints is regarded as a major factor in ROS generation.

Although isolated bouts of intense aerobic exercise have the potential to induce oxidative damage to muscle fibers, regular aerobic exercise improves the cellular capacity to detoxify accumulated reactive oxygen species (ROS) [53]. Frequent or moderate exercise has been shown to enhance antioxidant defense by increasing the activity of endogenous antioxidant enzymes such as catalase, GPx, and superoxide dismutase (SOD) [54]. Exercise protects the body against ongoing mild or moderate ROS exposure through redox-associated preconditioning, including repair processes for oxidative damage. In fact, long-term resistance and endurance training promote muscle mitochondrial proliferation, and the combination of both training modalities results in a more pronounced reduction in oxidative damage to lipids and carbohydrates, as well as a higher increase in antioxidant enzyme activity, indicating that combining the two types of exercise is beneficial in preventing type 2 diabetes [47]. Additionally, a study focused on untrained men revealed that all three types of training, including endurance training, resistance training, and concurrent training (a combination of endurance and resistance), can reduce oxidative stress [55]. The adaptation is mediated by moderate exercise and involves an increase in myocellular antioxidant capacity, which helps to reduce ROS levels. Results from marathoners showed that ROS generation peaked in the middle of the race, but then returned to baseline levels at the finish line, suggesting that an antioxidant defense mechanism may be activated after the run to mitigate oxidative stress [56]. Furthermore, Sahlin et al. demonstrated in ultra-endurance exercises that exercise increases ROS generation in isolated mitochondria from human muscle in a reversible manner without showing any signs of a harmful vicious cycle [57]. Using electron spin resonance spectroscopy (ESR) and plasma malonaldehyde (MDA) concentrations, another study investigated the relationship between delayed onset muscle soreness (DOMS), muscle function, and ROS after downhill running in sports performance such as trail running. Although a 15% gradient increased ROS generation, the rise occurred after the peak in DOMS and decreased muscle function, indicating that there may be a temporal decoupling between ROS, the loss of muscle function, and DOMS [58].

Continuing from the previous paragraph and specifically related to mountain sports performance, physical training at high altitudes can exacerbate oxidative stress, thereby playing a significant role in ROS generation. Hypobaric hypoxia, in particular, leads to a high number of ROS, resulting in potential tissue damage for mountaineers in the future [59]. In fact, a recent review has shown that ROS generation not only affects athletes but also a growing number of people who live or work at high altitudes with hypobaric hypoxia. This causes various changes in the body’s physiology, biochemistry, and molecules, leading to the development of hypoxic pulmonary vasoconstriction (HPV), remodeling, and pulmonary hypertension (PH) [60]. Additionally, hypoxia can activate several sources that produce reactive oxygen species and RNS, resulting in oxidative damage that can affect cellular performance and significantly decrease organ function. Moreover, it appears that both the enzymatic and non-enzymatic antioxidant systems are weakened by high altitudes. Recent findings indicate that the expression of Mn-SOD in the skeletal muscle of mountaineers who spent more than 6 weeks at an altitude of 6000 m dropped considerably one week after leaving the altitude. Furthermore, exposure to high altitudes increased the expression of Ku70, a gene involved in DNA repair, indicating increased DNA damage [53]. To combat this oxidative stress and maintain the balance between pro-oxidants and antioxidants, the human body has an antioxidant system that may be overwhelmed by increasing ROS levels but can be reinforced with antioxidant supplements [61]. However, inconsistency in the findings of numerous studies persists, likely due to variations in exercise regimens and assessment techniques for ROS in skeletal muscle.

## 5. Training Adaptation and Reactive Oxygen Species

### 5.1. ROS in the Skeletal Muscle

Reactive oxygen species (ROS) have conventionally been viewed as harmful constituents; however, it is now recognized that they are compelling signaling molecules that control physiological processes. Mitochondria in skeletal muscle cells typically generate ROS. Various conditions, including instances of strenuous contractile activity, can result in heightened mitochondrial ROS production during exercise [62]. Specifically, superoxide production in skeletal muscles can rise up to 50–100 times during aerobic exercise contractions [63]. ROS generated by mitochondria are a constant by-product of cellular metabolism, and their control is crucial for maintaining proper cellular function. Studies have demonstrated an atypical regulation of AMPK by mitochondrial ROS, which preserves cellular metabolic balance by prompting a PGC-1a-mediated antioxidant reaction. This response restricts mitochondrial ROS production and regulates HIF-1a stabilization, thereby enhancing stress tolerance and sustaining metabolic homeostasis [64].

However, recent findings indicate that mitochondria may not be the primary source of ROS during exercise, and further investigation is necessary to fully comprehend the involvement of mitochondria in ROS production during skeletal muscle contractions [62]. The reduced rate of superoxide generation can be attributed to uncoupling proteins, particularly UCP3, in skeletal muscle, which function as regulators of mitochondrial ROS production to prevent oxidative harm to mitochondria [65]. Additionally, emerging data suggest that mitochondria produce more ROS during state 4 (basal respiration) than during state 3 (maximal ADP-stimulated respiration) [66,67]. As skeletal muscle mitochondria are primarily in state 3 during aerobic contractions, their capacity to produce ROS during contractions is limited [66,67].

Heat shock proteins (HSPs) are known to play a critical role in safeguarding cells from various stress conditions, with exercise being a primary trigger for HSP expression. This expression can be prompted by a combination of stressors, in which ROS may exert a significant influence, as moderate levels of ROS serve as regulatory mediators in signaling pathways that shield cells from oxidative stress response [68].

Several studies have shown that high-intensity physical activity can result in oxidative stress, irrespective of the type of exercise, while other research has observed no significant changes in oxidative stress markers [69]. Follow-up investigations have investigated the effects of acute exercise and training on the body’s inherent antioxidant defense mechanisms. The findings demonstrate that acute exercise reduces the antioxidant capacity in the immediate post-exercise period but enhances it during the subsequent recovery period. Furthermore, these studies have revealed that physical training reduces ROS production during rest while boosting the body’s inherent antioxidant defense mechanisms [69].

Regular exercise not only offers systemic advantages to brain function by altering the cellular redox state but also enhances neurogenesis and cytokine production and reduces the accumulation of oxidative damage [53].

### 5.2. ROS in Endurance Training

The involvement of reactive oxygen species in the enhancement of performance- and health-related parameters induced by endurance training has a dual aspect. Endurance training performed regularly is advantageous and provides preventive effects against numerous diseases, delays symptoms of depression and neurodegeneration, and supports healthy aging by promoting mitochondrial biogenesis and oxidative metabolism. However, excessive exercise can hinder the adaptive process, leading to maladaptation and potentially an increased risk of developing diseases [70]. Investigations using cultured cells have demonstrated that ROS act as a trigger to induce mitochondrial biogenesis and increase the mRNA and protein expression of antioxidant enzymes [71].

On the other hand, the results of in vivo studies have been conflicting, and there is no consensus regarding the role of ROS as a trigger. Previous research suggests that ROS are involved in the stimulation of mitochondrial biogenesis induced by endurance training [72]. Other investigations have indicated that improved insulin sensitivity, as measured by the glucose infusion rate (GIR) and plasma levels of adiponectin, was only observed in the absence of antioxidants following endurance training [73].

Alternatively, a human-subject investigation was carried out to examine the impact of the administration of vitamins C and E on glycogen concentration, citrate synthase activity in skeletal muscle, maximal oxygen uptake, and endurance performance in response to endurance training [74]. The results showed that there was no effect on any of these parameters. Furthermore, the impact of antioxidant administration during rigorous endurance training on the enhancement of insulin-stimulated glucose uptake in healthy individuals was investigated [75]. The study revealed that the administration of antioxidants did not alter the improvement in glucose uptake induced by training.

Earlier research sought to examine the impact of training and/or antioxidant supplementation (vitamin E and alpha-lipoic acid) on skeletal muscle adaptations in rats [76]. Through an enhanced study design, it was discovered that antioxidant supplementation led to a reduction in the overall level of mitochondrial biogenesis, while still permitting the induction of mitochondrial biogenesis induced by endurance training. In essence, although antioxidant supplementation does not impede endurance training-induced mitochondrial biogenesis, it does result in a decrease in the overall level of mitochondrial biogenesis.

Endurance exercise triggers the activation of numerous intracellular signaling pathways, which promote mitochondrial biogenesis, angiogenesis, and heightened insulin sensitivity [77]. Earlier research has demonstrated that modifications in intracellular energy and calcium status during exercise function as the principal triggers. Contemporary investigations propose that ROS also serve as a stimulus during exercise. Additionally, the activation of the beta-adrenergic system and elevation of free fatty acid concentration have been identified as supplementary stimuli [78]. In this line, ultra-endurance exercise boosts the generation of mitochondrial ROS, which is linked to plasma-free fatty acid levels, yet it does not inevitably lead to oxidative harm to mitochondrial proteins [79].

To obtain a more comprehensive comprehension of the functions of ROS and antioxidants in exercise-induced adaptations, forthcoming investigations should consider the following suggestions. Initially, it is crucial to acknowledge that there are diverse sites of ROS production, which are reliant on the training conditions, such as intensity, duration, and environmental factors. Consequently, endeavors should be made to pinpoint the locations or compartments of ROS production and the repercussions of antioxidants on the redox status under each exercise condition [80,81]. To achieve this objective, it is advisable to evaluate multiple biomarkers of oxidative stress or redox status across various sites of ROS production. Secondly, it is essential to consider the redundancy of in vivo cellular signaling involved in adaptations induced by endurance training. Thus, forthcoming investigations should assess the relative importance and interconnections of signaling pathways emerging from distinct initial stimuli originating from exercise under every training condition [82].

### 5.3. Role of Reactive Oxygen Species (ROS) in Resistance Training

Resistance training (RT) can stimulate an increase in reactive oxygen species (ROS), which may result in muscle damage and fatigue. Antioxidant supplements are commonly ingested to mitigate these adverse effects, but recent studies suggest that ROS also play a significant role in cellular adaptation. Traditionally, the role of ROS in muscle damage and fatigue has been explored in the context of endurance training [83].

Consensus holds that heightened production of ROS has detrimental outcomes, as they are known to cause harmful effects. However, it should be acknowledged that ROS also have the ability to stimulate beneficial stress, known as eustress [84]. Therefore, ROS serve as important signaling molecules in redox regulation [85]. Nevertheless, uncontrolled and excessive production of ROS can lead to the formation of peroxides and damage cellular components such as proteins, lipids, and DNA [84,86]. In fact, it is believed that an increase in ROS generation is a contributing factor to the onset of degenerative diseases, aging, and muscle wasting [87,88].

Regarding RT, an 8-week study investigating the impact of endurance training, RT, and concurrent training on antioxidant capacity and oxidative stress markers in untrained individuals concluded that all groups demonstrated significant improvements in superoxide dismutase activity and noteworthy reductions in malondialdehyde levels, indicating that RT (at intensities greater than 50% of 1RM) brings about comparable changes in redox status as endurance training [55].

Middle-aged men (aged 53.9 ± 2.3 years) who underwent 21 weeks of resistance training twice a week showed greater upregulation of messenger ribonucleic acid (mRNA) levels of antioxidant enzymes such as catalase, GPX, and SOD compared to those who underwent endurance training [89]. Chronic resistance training offers protection against oxidative stress independent of training intensity. Both hypertrophy-intensity and strength-intensity training have been found to result in similar reductions in malondialdehyde levels and increases in glutathione levels in young men (aged 22.8 ± 2.7 years) [90]. These results have also been demonstrated in young, trained individuals [91], older adults [92], obese adults [93], and obese older women [94]. Sarcopenia, the age-related loss of muscle mass, strength, and function, may be triggered by the accumulation of reactive oxygen species (ROS), which cause oxidative stress and damage to cellular components, while exercise can help to delay or prevent sarcopenia despite increasing mechanical damage and accumulation of free radicals [95].

As a result, increasing evidence suggests that reactive oxygen species (ROS) may function as crucial intracellular signaling molecules that promote adaptations induced by resistance training (RT) to enhance cellular tolerance to future stress.

## 6. Reactive Oxygen Species, Antioxidants, and Inflammation

### 6.1. The Effect of Antioxidants on Reactive Oxygen Species in Physical Activity

Reactive oxygen species (ROS) are natural byproducts of cellular processes, especially during aerobic metabolism, and play a significant role in cell signaling. However, an imbalance between ROS generation and antioxidant presence can result in oxidative stress, leading to cellular and tissue damage, and contributing to various pathological conditions, including inflammation [96]. In recent years, antioxidant supplements have gained significant attention as a non-invasive approach to mitigate muscle damage, enhance exercise performance, prevent oxidative stress, improve lifespan and performance, and reduce the risk of adverse health effects associated with intense exercise in athletes [97].

Achieving an optimal antioxidant status can be achieved by consuming sufficient amounts of minerals and vitamins through a balanced and comprehensive diet that includes antioxidant-rich foods such as fruits, vegetables, and fiber. This approach ensures that antioxidants are present in natural ratios and proportions that can synergistically enhance their antioxidant effect [41,98,99,100]. Moreover, research has shown that quercetin supplementation may have beneficial effects on inflammation, maximal aerobic capacity, and fatigue during prolonged exercise in untrained but healthy individuals. A seven-day course of quercetin supplementation led to a slight increase in VO2max and a significant increase in ride time to fatigue, suggesting that quercetin may enhance endurance even without exercise training, which could be advantageous for athletic and military performance. Furthermore, the improvement in fitness without exercise training could have broader implications for health promotion and disease prevention [101].

Multiple studies conducted worldwide have consistently shown that a diet rich in vegetables, fruits, and legumes, which are abundant sources of antioxidants, is inversely related to inflammatory markers in the blood, including CRP, IL-6, and adhesion factors [102,103]. These findings are supported by another study that investigated adolescents aged 13–17 years and found that a diet rich in fruits and vegetables, which provide antioxidants, folate, and flavonoids, was associated with reduced levels of inflammation markers such as CRP, IL-6, and TNF-α [104]. Furthermore, a study reported that a three-week intervention with a 300 mg/d anthocyanin extract from blueberries resulted in a significant decrease in plasma concentrations of pro-inflammatory cytokines and chemokines associated with NF-kB signaling pathway activation, including IL-4, IL-13, IL-8, and IFN-a [105].

### 6.2. The Effect of Antioxidants on ROS and Inflammation in Sports

Altitude training is a common practice to enhance athletic performance by increasing oxygen-carrying capacity, but it is also associated with increased oxidative stress and inflammation. In a previous study, 31 elite endurance athletes were randomly divided into two groups during a three-week-long altitude training camp at 2320 m. One group consumed antioxidant-rich foods, while the control group consumed eucaloric foods. The results showed that the consumption of antioxidant-rich foods increased the athletes’ antioxidant capacity and partially reduced systemic inflammatory biomarkers associated with altitude training. However, the antioxidant intervention did not affect oxidative stress induced by altitude training or acute cytokine responses to exercise stress tests [106].

In contrast, a different study investigating the effects of consuming flavonoid-rich fresh fruit and vegetable juice on various biomarkers in elite sprint and middle-distance swimmers reported different findings. The results showed no significant effect of consuming juice for 10 days on chronic resting and post-exercise inflammation, oxidative stress, immune function, and metabolic profiles, and no significant benefits were observed other than an increase in nutrient intake [107].

The effects of different antioxidant supplements on exercise performance, antioxidant capacity, inflammation, and immune changes have been investigated in several studies. In one study, the influence of watermelon intake on exercise performance was studied in cyclists. The results showed that watermelon consumption supported the energy demands of exercise, increased post-exercise levels of nutritional components such as l-citrulline and l-arginine, and improved antioxidant capacity and total nitrate levels. However, it did not have a significant effect on post-exercise inflammation and innate immune function changes [108].

Another study investigated the effects of natural pomegranate juice (POMj) supplementation on performance and muscle soreness after weightlifting training sessions in elite weightlifters. The study found that consuming POMj supplementation reduced acute and delayed muscle soreness, inflammation, and damage, and improved the recovery kinetics of biological parameters, ultimately leading to improved weightlifting performance [109].

The impact of antioxidant supplements on inflammation was also assessed during intermittent shuttle running over a 6-week period in a study with 38 male participants. The study found that combined vitamin C and E supplementation did not reduce markers of oxidative stress or inflammation nor did it facilitate recovery of muscle function after exercise-induced muscle damage [110]. Furthermore, a study investigated the effectiveness of vitamin E and C supplementation in alleviating exercise-induced lipid peroxidation and inflammation in runners during a 50 km ultramarathon. The findings showed that markers of inflammation increased significantly in response to the run regardless of the treatment group, although antioxidant supplementation was found to prevent endurance-exercise-induced lipid peroxidation [111].

Finally, to assess the impact of a four-week course of antioxidant (AOX) supplementation on exercise-induced lipid peroxidation, muscle damage, and inflammation, kayakers were randomly allocated to receive either a placebo or an AOX capsule in a study with 20 participants. The results suggested that AOX supplementation did not confer protection against exercise-induced lipid peroxidation and inflammation and may impede muscle recovery [112].

In general, the efficacy of antioxidant supplements in mitigating inflammation and muscle damage and promoting recovery has yielded inconsistent outcomes due to discrepancies in exercise regimens, study methodologies, and analytical approaches. Further research is needed to better understand the role of antioxidants in exercise performance and recovery.

## 7. Antioxidant Capacity and the Microbiota

The gut microbiota refers to a group of microorganisms that reside throughout the gastrointestinal tract of mammals from the stomach to the colon. Its capacity is estimated to hold 10^14 cells (10 times the total number of cells in the human body), and yet, authors describe it as a symbiotic super-organism made up of eukaryotic and prokaryotic cells [113]. Composed of 500–1000 different species, its composition is host-specific, evolving throughout an individual’s life and influenced by both exogenous and endogenous factors. In this line, its composition begins to form at birth and continues to evolve during lactation. Babies delivered vaginally tend to have a more diverse microbiota dominated by Lactobacillus, Prevotella, or Sneathia species, while those born by Cesarean have less diverse microbiota with a predominance of Staphylococcus, Corynebacterium, and Propionibacterium. In childhood, the microbiota adopts the composition of adulthood, in which only 7–9 phyla of the total phyla that make up the Bacteria domain are represented. In the adult microbiota, 90% of the phylotypes belong to the Firmicutes (60–80%) and Bacteroidetes (15–30%) phyla, while the other minority groups belong to Proteobacteria, Actinobacteria, Fusobacteria, and Verrucomicrobia phyla [113].

Despite this chronic conformation, there are exogenous factors that can alter its composition, especially in adulthood. For example, stress and imbalanced diets are just two factors that can modify the composition of the gut microbiota, leading to a state of dysbiosis that may have negative effects on overall health [114]. Therefore, a stable and well-balanced gut microbiota is necessary to be in a state of homeostasis, capable of maximizing the beneficial interactions among its various members and with the host, which will help resist external and internal influences [115].

Among the major factors shaping the composition of the gut microbiota, physical activity and nutrition are two of them, with them influencing various mechanisms, such as the release of hormones and the redirection of blood from the gut to the skeletal muscles. In this line, the type, intensity, and duration of physical activity as well as the dietary intake of micro and macronutrients have a potential effect on the gut microbial population, ultimately altering its enzymatic potential and affecting the host’s overall health [116]. Yet, nutrition is the most powerful modulator.

In this line, macronutrients would be related to the supply of energy and structural support to the body, while micronutrients, such as vitamins, minerals, and other bioactive compounds, regulate biochemical processes and safeguard against various illnesses. These micronutrients play an important role in counteracting free radicals such as ROS and RONS that cause oxidative stress [117]. Among them, carotenoids, polyphenols, ascorbic acid, tocopherols, zinc, and selenium are present in food [118]; yet, supplementation may be necessary, especially in athletes as seen before. In this line, recent studies have shown that the gut microbiota, the microbes present in the intestine, are involved in vital biological processes such as regulating the immune system, nutrient absorption, and protecting against pathogens [119]. Interestingly, dietary antioxidants have been observed to modulate gut microbes while these same microbes produce useful metabolites by metabolizing the antioxidants. Thus, highlighting the importance and understanding of the interactions between dietary substances and gut microbes to develop therapeutic and preventive strategies targeting the gut microbiota is essential.

One of the most studied micronutrients is carotenoids, essential for humans because they act as precursors to vitamin A. They can be found in carrots, spinach, corn, tomatoes, and bell peppers, comprising compounds such as β-carotene, α-carotene, β-cryptoxanthin, lutein, zeaxanthin, neoxanthin, capsanthin, bixin, and lycopene. The presence of conjugated double bonds in carotenoids makes them act as antioxidants, which are a significant target of free radicals. In this line, researchers have demonstrated that carotenoids have preventive properties for cancer and several eye disorders, especially lutein and zeaxanthin [120].

Studies have shown that the consumption of β-carotene with other antioxidants such as tocopherol and selenium can decrease mortality rates in stomach cancer [121]. Lycopene, a vital antioxidant compound found in tomatoes, might cut the chance of upper aero-digestive tract cancer [122]. Astaxanthin consumption could greatly improve the lipid profile, leading to reduced low-density lipoprotein (LDL), cholesterol, and triglyceride levels [123]. A few studies have explored the interaction of different types of carotenoids with the gut microbiome. A study with a β-carotene oxygenase knockout mouse (C57BL/6 mice) model demonstrated that astaxanthin treatment significantly increased the population of Mucispirillum schaedleri and Akkermansia muciniphila compared to wild-type mice [124]. Similarly, in another pilot study, the administration of astaxanthin in the diet of β-carotene oxygenase knockout mice altered the gut microbiota, which was different in both genders of mice [125]. Studies on β-Carotene-15, 15′-oxygenase (BCO1)/β-carotene-9′, 10′-oxygenase (BCO2) double knockout mice models revealed that a meal supplemented with tomato powder boosted the prevalence of helpful microorganisms such as Lactobacillus and Bifidobacterium [126].

Other antioxidants largely studied are polyphenols. These are aromatic molecules consisting of phenolic rings and are further classified into various subgroups, including flavonoids, phenolic acids, stilbenes, and lignans [127]. Major sources of polyphenols are blueberries, raspberries, oranges, onions, soybeans, and green tea leaves. Over the years, polyphenols have been the focus of several epidemiological cohort and case-control-based studies to determine their effects on human health. Researchers suggest that a polyphenol-rich diet may protect against chronic diseases such as cancer, cardiovascular diseases, and inflammatory diseases [128]. Furthermore, there is a subgroup of polyphenols called flavonoids, which can be classified into various types, including flavonols, flavones, isoflavones, flavanones, anthocyanidins, and flavanols. These molecules exhibit pleiotropic properties, including anti-inflammatory, antiallergenic, antiviral, neuroprotective, cardioprotective, and chemoprotective properties [129]. Researchers have found that flavonoids scavenge oxygen-free radicals or inhibit the enzyme responsible for producing ROS; thus, the catechol group of flavonoids plays a crucial role in free radical scavenging and antioxidant activity [130]. Furthermore, anthocyanins, another subgroup of flavonoids possess significant antioxidant activity. Some of the different anthocyanins include delphinidin, petunidin, malvidin, cyanidin, peonidin, and pelargonidin. Researchers attribute them to anticancer, antimicrobial, and protective properties against cardiovascular diseases [131]. In this line, studies have suggested the effectiveness of anthocyanin-rich diets in significantly reducing fat accumulation in the body, and this could be possible because anthocyanins attenuate the synthesis of lipids in adipose tissue and the liver [132]. Additionally, studies have shown that consuming antioxidant-rich bilberry extracts significantly reduces blood sugar and increases insulin sensitivity, which is crucial in diabetic conditions [133].

Polyphenols also interact with gut microbiomes, and gut microflora plays a vital role in the metabolism of dietary polyphenols. Dietary polyphenols undergo conjugation in the small intestine and are then sent to hepatocytes for phase I and II metabolisms. Water-soluble conjugates of polyphenol molecules travel through the small intestine and the systematic circulation to several organs and are finally excreted through urine. The unabsorbed molecules undergo several enzymatic alterations such as hydrolysis, reduction, dehydroxylation, decarboxylation, demethylation, and ring fission, mainly carried out by gut microbes in the lumen of the large intestine [134]. Thus, their pleiotropic attributes make them attractive agents for disease prevention and treatment. Their consumption in a diet or throw supplementation may be a useful protection against various chronic diseases. Yet, further research is necessary to determine the specific mechanisms of polyphenols in promoting human health.

Another largely studied micronutrient with antioxidant properties is vitamin C, also known as ascorbic acid. A water-soluble compound found in fruits such as oranges, lemons, grapefruit, and guava, it plays many roles, including acting as an antioxidant, a cofactor for enzymes, and supporting the immune system [135]. Indeed, it is essential since it regulates various transcription factors and signaling cascades, especially those related to the immune system and resistance against pathogens [136]. Its implication related to the gut microbiota is that vitamin C is absorbed from the intestine and stored in cells as ascorbic acid. In this line, pathogenic strains can decompose ascorbic acid, while some bacteria can reduce dehydroascorbic acid to ascorbic acid. Thus, vitamin C can positively modulate the gut microbiota and improve butyrate production, which contributes to restoring gut microbiota homeostasis and immune balance in ethanol-induced liver injury [135].

Vitamin E is a compound that includes tocopherols and tocotrienols, which are classified into subgroups based on the placement of methyl groups. Vitamin E is found in wheat germ oil, almonds, avocado, and various nuts [137]. Vitamin E has non-polar properties and acts as an antioxidant, protecting against free radical activity. Consuming vitamin E, along with vitamins C and carotene, can help protect against cardiovascular diseases. Vitamin E also helps protect against neurodegenerative disorders and cognitive impairment. Vitamin E facilitates the release of prostacyclin, which inhibits platelet aggregation and provides defense against viral infection [138]. Vitamin E improves immune system function and minimizes infection risk, especially in the elderly. Vitamin E interacts with gut microbes, which could be the reason for the health benefits exhibited by vitamin E. Vitamin E administration can modulate gut microbes and be helpful in colitis treatment. However, an excess amount of vitamin E amendment can increase the population of pathogenic bacteria, leading to various metabolic disorders [139].

Zinc is an essential mineral that plays a crucial role in human health. According to the World Health Organization, zinc deficiency affects around 2 billion people and can cause growth retardation, oxidative stress, and inflammation. Zinc is found in various food sources, such as lean meat, eggs, seafood, beans, nuts, and chickpeas. Additionally, zinc has been shown to be effective in treating diarrhea and reducing the duration of the common cold. Zinc’s role in human health is multifaceted [140]. It competes with iron and copper ions, restricting the development of free radicals that can damage cells. Zinc also promotes the synthesis of antioxidant proteins and enzymes while inhibiting oxidant-promoting enzymes, making it essential for maintaining a healthy immune system [141]. Supplementation of zinc in older adults has been found to reduce infections, and studies have shown that zinc supplementation can reduce various oxidative stress markers in plasma [142]. The interaction of zinc with the gut microbiome is an area of growing interest. Studies have shown that dietary zinc has a modulatory effect on gut microbial populations, with zinc-fortified diets increasing the population of beneficial microbes such as Lactobacillus reuteri, Dorea, Clostridiales, Ruminococcus, and Lachnospiraceae [143]. However, some studies have shown that exposure to zinc can reduce the population of beneficial gut microbes, such as Actinobacteria and Lactobacillus, and disturb the gut microbial community [144].

In line with these micronutrients, lastly, we can find selenium, an essential trace element required for the human body’s proper functioning [145]. This element is primarily found in seafood, cereals, and dairy products. Research studies have shown that seleno-proteins, which require selenium for their synthesis, are effective in reducing the oxidative stress generated by ROS and RONS. Selenium deficiency leads to congestive cardiomyopathy, also known as Keshan disease [146]. However, selenium is proven to improve the immune system, regulate thyroid hormones, and exhibit anticancer properties [147]. Apart from the role of selenium in maintaining human health, its interaction with the gut microbiome is an emerging area of research. A recent study has demonstrated that gut microbes play an active role in selenium metabolism [148]. Various microbes present in the human gut require selenium to express their selenoproteins, and dietary selenium is primarily found in two forms: Se-methionine and selenite [149]. The digestibility of Se-methionine is higher than selenite, and the absorption and metabolism of Se-methionine occur through the same pathways as methionine. Studies have shown that the gut microbiota-targeted therapeutic diets with polysaccharides are effective in treating obesity and diabetes [150]. Selenium-rich Cordyceps militaris polysaccharides (Se-CMP) have been shown to decrease serum LDL and increase beneficial gut bacteria such as Akkermansia [151]. Furthermore, a selenium-rich diet has been shown to increase the richness and diversity of gut microbes and increase beneficial microbial species such as Christensenellaceae, Ruminococcaceae, and Lactobacillus [152]. The authors of the study also elucidated that the increase in glutathione peroxidase and selenoalbumin after the administration of a selenium-rich diet could be related to the positive changes in gut microflora. These findings suggest that selenium and gut microbes have an active bi-directional interaction [153].

## 8. Antioxidants and Recovery from Training

Antioxidants play a crucial role in aiding an athlete’s recovery after training. During exercise, there is increased release of free radicals and reactive oxygen species (ROS) by the body, which can damage cells and cause oxidative stress in tissues [154]. This rise in ROS has been established as a trigger for muscle damage (DOMS) and reduced performance [155]. However, ROS is produced in various biological processes and can signal the body’s response to physical activity for proper adaptations [156].

Despite the health benefits of physical activity, it can also lead to an increase in oxidative stress, resulting in higher production of free radicals and decreased antioxidant defenses in the body [25]. High-intensity and eccentric exercises, in particular, have been found to reduce antioxidant defenses and cause muscle damage, leading to DOMS [157]. However, recent studies have shown that ROS induced by physical exercise can also regulate enzymatic and non-enzymatic antioxidants in the body, protecting against cellular oxidative damage and optimizing signaling for proper muscle adaptation [158]. Nevertheless, intense physical exercise can also impact the immune and hormonal systems, leading to increased levels of interleukin 6 (IL-6), creatine kinase (CK), glutamic oxaloacetic transaminase (GOT), and glutamic-pyruvic transaminase (GPT) [159].

DOMS typically peaks between 24 to 72 h after a workout and gradually subsides within 5 to 7 days [160]. As a result, athletes have had to adopt strategies to reduce acute muscle damage and hasten recovery for subsequent training sessions. Among the various recovery strategies, antioxidant supplementation has become a common practice among athletes, as these molecules neutralize ROS and help prevent damage [161]. Ingesting supplements or foods with a high antioxidant content in doses higher than the recommended levels in the days leading up to sports competitions has been reported to prevent or reduce DOMS [162]. Antioxidants are generally classified as exogenous (such as vitamin C, vitamin E, polyphenols, glutathione, carotenoids, and coenzyme Q10) and endogenous (including plasma proteins, bilirubin, uric acid, and the enzymes superoxide dismutase, glutathione peroxidase, and catalase) [161]. Numerous studies have investigated the efficacy of antioxidants (from whole natural food sources, antioxidant extracts, or vitamin C and E supplements, among others) in alleviating muscle pain and soreness in various exercise modalities, yielding controversial results.

In the context of strength training, Ammar et al. [109] conducted a study that showed an improvement in recovery from oxidative stress with pomegranate juice compared to a placebo after two training sessions involving Olympic exercises (snatch, clean and jerk, and squat). The authors observed a reduction in malondialdehyde (MDA), which is known to have a significant effect on lipid peroxidation and antioxidant parameters. Similarly, Leonardo-Mendoça et al. [163] found that the supplementation of 100 mg of melatonin per day protected skeletal muscles against oxidative damage in well-trained athletes. This was evident through a reduction in CK, lactate dehydrogenase (LDH), creatinine, and total cholesterol levels after 4 weeks of consumption compared to the placebo.

In another study, melatonin supplementation of 20 mg per day during two weeks of high-intensity interval training (HIIT) and strength training showed improved antioxidant status in highly trained athletes. This was reflected in increased melatonin levels and glutathione peroxidase activity, as well as reduced muscle DNA damage [164]. Moreover, Stefan et al. [165] observed an improvement in perceived muscle recovery and a reduction in serum CK levels after 5 weeks of full-body training exercises in both males and females aged 21 to 65 years following L-carnitine supplementation. Regarding vitamin supplementation, Silva et al. [166] demonstrated that vitamin E supplementation at a dose of 800 IU of D-α-tocopherol acetate per day for 21 days resulted in significantly lower levels of muscular damage and oxidative stress compared to the control group. This was assessed after three sets of flexion and extension of the elbow on the Scott bench at 2 min intervals until exhaustion. However, there was no significant difference in the inflammatory response (TNF-α and IL-10) between the two groups. Furthermore, de Oliveira et al. [167] reported that antioxidant vitamin supplementation effectively prevented oxidative stress in soccer athletes through a reduction in malondialdehyde lipid peroxidation, total lipid peroxidation, and the ratio of glutathione to oxidized glutathione after consumption of VitC (500 mg) and VitE (400 IU of α-tocopherol) per day for 15 days. However, this vitamin supplementation did not significantly reduce the plasma CK concentration or DOMS during the recovery days after an acute bout of plyometric jump and strength resistance set to exhaustion. Despite this, a recent systematic review [159] established that adequate supplementation with antioxidants can help reduce the oxidative stress generated by resistance training.

In the context of aerobic training, Zhang et al. [168] conducted a study that showed the positive effects of oats on the body. They found that a daily dose of 20 mg of oats reduced circulatory inflammatory cytokines and CK levels after 8 weeks of downhill running (10% grade with an intensity equivalent to 75% of their maximal heart rate) in active participants. Similarly, Drobnic et al. [169] observed a decrease in interleukin 8 (IL-8) values, resulting in reduced muscular trauma and moderate pain in the posterior and medial thigh after a downhill run (test on a treadmill at grade -10% at a constant speed with an intensity of aerobic threshold for 45 min), following the consumption of 200 mg of curcumin twice a day for 5 days prior to the test. Another study by He et al. [170] showed that an antioxidant supplement (100 mg vitamin C and 400 IU vitamin E ingested daily for 2 weeks) reduced muscle soreness in the quadriceps and tibialis anterior, as well as CK levels, and increased activity and oxygen radical absorbance capacity after the first bout of exercise (40 min of downhill running at −10% grade at 65% to 70% VO2max). However, these parameters were not significantly different between the antioxidant and placebo groups in a second session, indicating that antioxidant supplementation did not prevent the adaptation that occurs with repeated sessions. On the other hand, Close et al. [171] found that ascorbic acid supplementation (1.5 g of ascorbic acid daily for three days before and two days after the trial) did not reduce muscle soreness compared to the placebo group after a bout of exercise designed to cause muscle damage (downhill running on a motorized treadmill for 30 min). However, elevated serum MDA levels were reported in the placebo group only after 72 and 96 h post exercise. Similarly, Kashef et al. [172] showed that vitamin E supplementation (400 IU per day for 30 days) had no significant effect on the development of DOMS in young men after 10 min of bench leg step ups with both legs. There were no significant differences between the vitamin E and placebo groups in terms of VAS scores and CK and CRP levels.

Despite the findings mentioned above, different reviews with meta-analyses have concluded that there is little evidence to support the use of antioxidants for preventing or reducing muscle soreness after exercise [161,173,174]. This is because the recovery process is a complex phenomenon that can be influenced by many variables, such as differences in the antioxidant dosage, the length of supplementation, the type of participants, or differences in size, body weight, and body composition, which may result in different responses to antioxidant supplementation. Therefore, further research is needed to confirm these findings and investigate the potential long-term effects of antioxidant supplementation on exercise recovery against acute effects.

## 9. The Effect of an Antioxidant Diet on Sports Performance

Exercise-induced oxidative stress can lead to muscle damage, impaired performance, and fatigue. During exercise, there is an increase in oxygen consumption, resulting in the production of reactive oxygen species (ROS) [154]. These ROS can cause damage to cells and tissues, leading to oxidative stress. As a result, oxidative stress plays a role in the development of fatigue and may impair performance in athletes. However, antioxidants, which are compounds that can neutralize free radicals and reduce oxidative stress, can have a positive impact on athletic performance. Several studies have examined the impact of antioxidants on various aspects of sports performance, such as endurance, strength, and recovery [96]. Despite some suggestions in the scientific community that certain antioxidants may have a positive impact on athletic performance, the evidence is not strong enough to make specific recommendations for athletes [174]. Therefore, implementing an antioxidant-rich diet may reduce oxidative stress and improve exercise performance in this population. However, the optimal dose, duration, and timing of antioxidant supplementation remain unclear, and more research is needed to determine the potential benefits and risks of antioxidant supplementation for athletes [161].

One simple and effective way to maintain an antioxidant status that helps reduce the oxidative stress generated by physical exercise is through the addition of foods rich in antioxidants to a balanced diet, such as the Mediterranean diet [175]. Adhering to a Mediterranean-style diet has been shown to enhance antioxidant defenses, improve lipid profile, and reduce LDL oxidation more than a high-fat diet [11]. However, there have been limited studies conducted to specifically examine the antioxidant effects of a particular diet on sports performance.

Therefore, while some evidence suggests that an antioxidant-rich diet may positively impact athletic performance, further research is needed to establish clear recommendations for athletes. It is important to consider various factors such as the type of antioxidant, dosage, duration of supplementation, and timing in relation to exercise, as well as individual differences in size, body weight, and body composition that may influence the response to antioxidant supplementation. In conclusion, while antioxidants may have potential benefits for athletes, more research is needed to fully understand their effects on sports performance and recovery.

In the context of antioxidant-rich diets, various studies have investigated the effects of specific antioxidant compounds on athletic performance. For instance, Gómez-Cabrera et al. [72] found that vitamin C supplementation (1 g/day) led to a smaller increase in muscle mitochondrial biogenesis and a smaller improvement in endurance performance compared to placebo after an 8-week supervised resistance training program on a cycle ergometer. However, Braakhuis et al. [176] reported that blackcurrant (BC) juice (15 mg VC, 300 mg anthocyanins) slightly increased the maximum running speed in an incremental trial more than vitamin C (1 g) alone and the placebo after 3 weeks of high-intensity training. Similarly, Aguiló et al. [177] demonstrated that 90 days of supplementation with antioxidants (500 mg/day of vitamin E, 30 mg/day of β-carotene, and 1 g/day of vitamin C in the last 15 days) resulted in a significant improvement in aerobic performance (7.2% increase in VO_2_ max) and a lower peak blood lactate concentration compared to the placebo group after a maximal exercise test on a cycle ergometer. However, Paulsen et al. [178] reported that vitamin C and E supplements (1000 mg and 235 mg for 11 weeks, respectively) had less improvement in endurance performance compared to the placebo, suggesting that high doses of vitamins may interfere with cellular adaptations that occur in response to endurance training, particularly in the muscles (Table 1). Hence, athletes should be cautious about high doses of antioxidant supplements, as they may potentially decrease performance, as reported in various studies [96].

In the case of quercetin, Daneshvar et al. [179] showed that supplementation with 1000 mg of quercetin per day for eight weeks resulted in significant improvements in exercise performance (a 5.2% increase in cycle time in an incremental trial) compared to the placebo in male badminton players. Similarly, MacRae et al. [180] found that a combination of quercetin and antioxidant supplements led to a significant improvement in cycling time trial performance compared to the placebo, quercetin alone, or isolated antioxidants in elite male cyclists. However, Darvishi et al. [181] reported no significant difference in swim performance or body composition between the quercetin supplementation group (1000 mg) and the placebo group in female swimmers. Additionally, the supplementation of 1000 mg of quercetin per day for 1 week did not show significant differences in VO_2_ max and race time relative to the placebo in the untrained participants [182].

In relation to resveratrol, there have been limited studies conducted in human models compared to animal models, and the effects of this supplement on performance parameters remain controversial [11]. For instance, Gliemann et al. [183] found that exercise training combined with resveratrol supplementation (250 mg per week for 8 weeks) did not result in the same magnitude of improvement in performance and health parameters (such as maximal oxygen uptake, blood pressure, and cholesterol levels) as exercise alone in elderly untrained males.

As for beetroot juice, Wylie et al. [184] reported that supplementation with 140 mL of beetroot juice resulted in significant increases in plasma nitrite levels compared to a placebo. Moreover, beetroot juice also led to a significant increase in power output during the last 10 min of an incremental cycle ergometer test in young men. Similarly, Lansley et al. [185] investigated the effect of beetroot juice consumed 2.5 h prior to completing a 4 km cycling time trial in elite male cyclists. The authors found that the cyclists completed the time trial significantly faster after consuming the beetroot juice supplement compared to the placebo supplement. The average power output during the time trial was also significantly higher after consuming the beetroot juice supplement. Furthermore, Wylie et al. [186] demonstrated that the group consuming nitrate-rich beetroot juice (280 mL per day for 2 days) had significantly improved performance compared to the placebo group. Specifically, the nitrate supplementation led to an increase in the distance covered in a Yo-Yo Intermittent Recovery Level 1 test in team sport players. However, Hoon et al. [187] reported in their meta-analysis that nitrate supplementation might have a beneficial effect in non-athletes but concluded that athletes do not respond to this supplementation.

## 10. The Effect of Antioxidant Supplementation on Sports Performance

Antioxidant supplementation has been widely used in sports to improve performance and prevent cell and tissue damage caused by oxidative stress during intense exercise [13]. During intense exercise, there is an increase in free radical production, which can contribute to muscle damage and fatigue [188]. Antioxidant supplementation may help counteract this effect by neutralizing free radicals and protecting cells and tissues. However, the results of studies in this area are mixed and inconclusive, with some sports showing benefits in antioxidant capacity and a reduction in muscle damage and inflammation, while others do not [188].

One of the most extensively studied antioxidants is vitamin C. Vitamin C supplementation has been suggested to improve athletic performance due to its antioxidant effects, its ability to regenerate other antioxidants, and its potential to improve immune function [13]. Some studies have found that vitamin C supplementation may improve antioxidant capacity, reduce muscle damage, and reduce inflammation in athletes [12]. However, the results are mixed and further studies are needed to fully assess the effect of vitamin C supplementation on exercise performance. For example, one study concluded that vitamin C may have a positive antioxidant effect but does not directly improve athletic performance in marathon runners [176]. Similarly, it has been shown that vitamin C supplementation does not improve performance in terms of race time or speed in long-distance cyclists. However, cyclists who took vitamin C experienced a significant reduction in muscle damage and inflammation after exercise [189]. On the other hand, vitamin C supplementation has been found to significantly reduce muscle damage and inflammation after exercise and may help improve muscle recovery after intense exercise [12].

Vitamin E supplementation may also be an effective strategy for improving athletic performance and reducing the oxidative stress associated with intense exercise. Vitamin E acts as an antioxidant, reducing oxidative damage to muscle cells and improving immune function and the regulation of lipid metabolism [190]. Overall, vitamin E supplementation appears to have a beneficial effect on exercise performance by reducing oxidative stress and muscle damage after exercise [13]. However, the results are mixed and further studies are needed to determine the exact effects of vitamin E supplementation in different sports and populations. For example, a meta-analysis of 22 trials found that vitamin E supplementation significantly improved exercise performance compared to a placebo [191]. Vitamin E supplementation was also associated with a reduction in inflammation and soreness after exercise [191]. In a study of endurance athletes, vitamin E supplementation for 8 weeks significantly increased antioxidant capacity and reduced muscle damage after exercise [72]. Another study in cyclists found that vitamin E supplementation for 4 weeks improved performance in an endurance test [192]. However, some studies have found no significant benefit of vitamin E supplementation on exercise performance, such as a study of football players which found no significant differences in performance after 8 weeks of vitamin E supplementation [193]. Table 2 shows the content of vitamin E in some foods.

There are several polyphenolic compounds found in various foods that have been studied for their potential effects on athletic performance. Resveratrol, found in grapes, blueberries, and peanuts, acts as an antioxidant, reducing oxidative damage to muscle cells and improving mitochondrial function, which is crucial for energy production and muscle function [194]. Some studies suggest that resveratrol supplementation may have beneficial effects on athletic performance by improving antioxidant capacity and reducing muscle damage, although the results may not be consistent across all studies or sports [195]. For example, a study in rats found that resveratrol supplementation improved performance in a swimming test and reduced oxidative stress in muscles [196], while another study in humans found that resveratrol supplementation improved plasma antioxidant capacity and reduced muscle damage after high-intensity exercise [197]. However, other studies have found no significant effect of resveratrol supplementation on exercise performance, such as one study of elite cyclists that found no improvement in a long-duration endurance test [198] and another study in football players that found no significant effect on physical or cognitive performance [199].

CoQ10, another compound with potential performance-enhancing effects, may improve athletic performance due to its antioxidant and energetic properties. CoQ10 plays a role in the mitochondrial electron transport chain, and studies suggest that supplementation may improve exercise capacity, reduce oxidative stress, shorten running time, and improve muscle recovery in athletes [195]. For example, a study of marathon runners found that CoQ10 supplementation reduced muscle damage and improved plasma antioxidant capacity after the race [200], and another study of elite cyclists found that CoQ10 supplementation improved performance in a long-duration endurance test [201]. In addition to its antioxidant effects, CoQ10 has also been proposed to enhance athletic performance by improving mitochondrial function and energy production. A pilot study of swimmers showed that CoQ10 supplementation improved energy efficiency during exercise and reduced muscle fatigue [202]. However, other studies have found no significant effect of CoQ10 supplementation on exercise performance, such as one study of swimmers that found no significant difference in performance after 18 sessions of CoQ10 supplementation [203], and another study in cyclists that found no significant effect on performance during a short endurance event [204,205].

Selenium, as a cofactor for glutathione peroxidase and its role in protein synthesis and the immune response, has been studied for its potential effects on athletic performance. While there is evidence to suggest that selenium supplementation may have beneficial antioxidant effects in the body, which could help improve the antioxidant response and reduce muscle damage and inflammation after intense exercise, there is no conclusive evidence that selenium supplementation directly improves exercise performance [206]. For example, one study showed that selenium supplementation improved antioxidant response in endurance athletes but did not result in significant improvements in performance [174], while another study showed improved thyroid function and antioxidant response in endurance athletes, but no significant improvements in exercise performance [207]. A recent study demonstrated improved muscle recovery after intense exercise through reduced muscle damage and inflammation [206]. However, more research is needed to determine the long-term effects of selenium supplementation on exercise performance and overall health.

Curcumin, known for its antioxidant effects and its potential to improve mitochondrial function and ATP production, has also been studied for its effects on athletic performance. Studies suggest that curcumin supplementation may improve exercise capacity, reduce oxidative stress, and reduce exercise-induced muscle soreness in athletes [208]. For example, one study showed that curcumin supplementation significantly reduced levels of muscle damage and inflammation after endurance exercise in young men, suggesting an antioxidant and anti-inflammatory effect [209]. Another study demonstrated that curcumin supplementation improved performance in terms of the distance covered and time taken during endurance exercise in cyclists compared to a placebo [169]. Furthermore, another study showed that curcumin supplementation significantly reduced levels of muscle damage and inflammation after endurance exercise in young women, indicating its potential antioxidant and anti-inflammatory effects. Additionally, curcumin supplementation was found to significantly improve performance in terms of time spent during endurance exercise when compared to a placebo [210].

In general, antioxidants such as vitamin C, vitamin E, resveratrol, coenzyme Q10, selenium, and curcumin have been shown to have positive effects on health and exercise performance. These antioxidants can help reduce inflammation, protect cells from oxidative damage, and improve muscle recovery after intense exercise. However, the specific effects may vary depending on the type of exercise, the dose and duration of supplementation, and individual characteristics. It is important to note that antioxidant supplementation should not replace a balanced diet and adequate hydration. Additionally, antioxidant supplementation should be individualized and supervised by a health professional, especially in people with chronic diseases or who are taking other medications.

## 11. Key Points and Strategies for Antioxidant Supplementation in Sports Competitions

### 11.1. Vitamin C

Vitamin C supplementation is widely used among athletes due to its antioxidant effects and potential to improve muscle recovery after intense exercise. The recommended dose of vitamin C for athletes varies depending on the duration and intensity of exercise. Typically, a daily dose of 500 to 2000 mg of vitamin C is recommended for maintaining good health and reducing oxidative stress. However, some studies suggest that higher doses (up to 3 g/day) may be beneficial for athletes who engage in intense and prolonged exercise, such as marathons, to prevent muscle damage and improve recovery [178]. Research also suggests that the timing of vitamin C supplementation may play a role in its effectiveness. Pre-exercise vitamin C supplementation has been shown to reduce exercise-induced muscle damage and inflammation [72]. On the other hand, post-exercise vitamin C supplementation may improve muscle recovery and reduce soreness [171]. The duration of vitamin C treatment in athletes varies based on training goals and individual needs. Generally, it is recommended to take vitamin C continuously for several weeks prior to competition to reduce oxidative stress and improve muscle recovery. Some studies suggest that vitamin C supplementation for 4–6 weeks prior to competition may enhance performance in high-intensity, short-duration exercise [176].

In conclusion, vitamin C supplementation in athletes may be beneficial in reducing oxidative stress and improving muscle recovery after intense exercise. Recommended doses may vary depending on the duration and intensity of exercise, and the timing of supplementation, either before or after exercise, may influence its effectiveness. The duration of treatment should be tailored according to training goals and individual needs. It is important to consult with a healthcare professional for personalized recommendations and to closely monitor for any potential side effects.

### 11.2. Vitamin E

Vitamin E supplementation in athletes has been studied for its potential effect in preventing exercise-induced muscle damage and improving athletic performance. The recommended dose of vitamin E supplementation for athletes varies depending on the duration and intensity of exercise and should not exceed 1000 IU per day to avoid possible adverse effects, such as reducing the body’s ability to clot blood. Vitamin E supplements can be taken before and after exercise. In one study, athletes were given 400 IU of vitamin E for 5 days before long-distance running, and supplementation was found to reduce the amount of DNA and protein oxidation products in skeletal muscle after exercise [211]. In another study, a single dose of 800 IU of vitamin E was given to subjects 30 min before endurance exercise, and a significant reduction in the levels of markers of muscle damage was observed compared with the placebo group [212]. The duration of vitamin E supplementation varies from study to study, with athletes receiving 400 IU of vitamin E for 5 days in one study [211], while in another study, a single dose of 800 IU of vitamin E was administered [212]. In conclusion, vitamin E supplementation may have beneficial effects in preventing exercise-induced muscle damage and improving athletic performance in athletes. However, the recommended dose of vitamin E supplementation varies according to the duration and intensity of exercise, and care should be taken not to exceed 1000 IU per day to avoid potential adverse effects. In addition, the duration of supplementation also varies from study to study, although it is generally recommended that vitamin E supplementation should be acute rather than chronic. More research is needed to better understand the role of vitamin E in health and exercise performance.

### 11.3. Resveratrol

Resveratrol is a polyphenol found in certain foods such as grapes, blueberries, and walnuts and has been studied for its potential to improve athletic performance through its antioxidant and anti-inflammatory effects. The recommended dose of resveratrol for improving athletic performance varies from study to study, but a daily dose of 250–500 mg has generally been used [213]. Resveratrol supplementation has been used in both the pre-competition and recovery phases. The duration of supplementation with resveratrol to improve athletic performance varies from study to study, but generally a duration of four to eight weeks has been used [214]. In conclusion, resveratrol supplementation may improve athletic performance through its antioxidant and anti-inflammatory effects. A daily dose of 250–500 mg is recommended for four to eight weeks, both in the pre-competition and recovery phases. However, further studies are needed to confirm these effects and to determine the optimal dose and duration of resveratrol supplementation in athletes.

### 11.4. Coenzyme Q10

CoQ10 supplementation has been shown to have positive effects on cardiovascular function, brain health, and antioxidant protection. Some studies have also investigated its potential effects on exercise performance in athletes. The recommended dose of CoQ10 varies from study to study, but a daily dose of 100–300 mg has generally been used to improve performance in athletes [215]. CoQ10 supplementation has been used in both the pre-competition and recovery phases and has been shown to be effective in both phases [215]. The duration of treatment also varies from study to study, but generally a duration of 4–12 weeks has been used [215]. A study investigated the effects of CoQ10 supplementation in elite Japanese kendo athletes. The participants were given 300 mg of CoQ10 daily for 20 days. Significant reductions in muscle damage and improvements in performance were observed compared to the control group [216]. Another study looked at the effects of CoQ10 supplementation in untrained males and females. The participants were given 100 mg of CoQ10 daily for 14 days before undergoing different tests [201]. Significant improvements in time to exhaustion and reductions in muscle damage were observed compared to the control group.

In conclusion, CoQ10 supplementation may improve athletic performance and reduce muscle damage. The recommended dose varies from study to study, but generally a daily dose of 100–300 mg for 4–12 weeks has been used, and supplementation can be taken in both the pre-competition and recovery phases.

### 11.5. Selenium

Selenium is an essential trace element for human health and plays an important role in the body’s antioxidant function. Selenium supplementation has been shown to improve general health and may have specific benefits for athletes. The recommended dose of selenium for athletes varies from study to study, but a daily dose of 200–400 mcg has generally been used. A recent systematic review and meta-analysis concluded that selenium supplementation at doses between 100–400 mcg/day may improve athletic performance in terms of aerobic capacity and muscle strength [206]. Selenium supplementation has been administered at various times, including before exercise, during exercise, and after exercise. The duration of selenium treatment also varies from trial to trial, but generally 4–12 weeks has been used. In general, selenium supplementation can be beneficial for athletes, but it is important to note that excess selenium can be toxic. It is recommended that daily doses do not exceed 400 mcg to avoid potential adverse effects [217].

### 11.6. Curcumin

Curcumin, a bioactive compound found in turmeric, has been extensively studied for its anti-inflammatory and antioxidant properties, making it a potential athletic-performance-enhancing supplement. The recommended dosage of curcumin for athletes varies depending on the purpose of supplementation. To reduce muscle soreness and improve recovery, doses of 400–500 mg of curcumin twice daily have been used. To improve athletic performance, doses of 90–500 mg of curcumin per day have been used [208]. Its supplementation can be taken either before or after exercise. Pre-exercise supplementation has been shown to reduce exercise-induced muscle inflammation and soreness [169]. Post-exercise supplementation may improve muscle recovery and reduce muscle soreness [218]. The duration of curcumin supplementation depends on the purpose of the supplementation. For reducing muscle soreness and improving recovery, treatments of 4–12 weeks have been used. Treatments of 2–8 weeks have been used to improve athletic performance [208]. In this line, a study investigated the effects of curcumin supplementation in women with moderate physical activity levels. The participants received 500 mg/day of curcumin for 8 weeks, and a significant reduction in muscle soreness and improved recovery was observed compared to the placebo group [219]. Another study investigated the effects of curcumin supplementation in healthy, moderately active men. The participants received 200 mg of curcumin twice daily for 2 days before and 1 day after an eccentric muscle injury protocol. A significant reduction in levels of inflammatory markers and muscle soreness was observed compared to the placebo group [169].

In conclusion, curcumin supplementation may be beneficial in reducing muscle soreness, improving recovery, and possibly improving athletic performance in athletes. Recommended doses vary depending on the purpose of supplementation, but doses of 400–500 mg twice daily have been used to reduce muscle soreness and improve recovery, and doses of 90–500 mg daily have been used to improve exercise performance. Supplementation can be taken either before or after exercise, and the duration of treatment varies depending on the purpose of supplementation, but 4–12-week courses have been used to reduce muscle soreness and improve recovery, and 2–8-week courses have been used to improve sports performance.

### 11.7. Omega-3

Omega-3 fatty acids, particularly eicosapentaenoic acid (EPA) and docosahexaenoic acid (DHA), are known for their anti-inflammatory and antioxidant properties, making them a potential supplement for athletes. Oxidative stress, which occurs when there is an imbalance between the production of reactive oxygen species (ROS) and the body’s ability to remove them, can negatively impact athletic performance and recovery. Omega-3s can help reduce oxidative stress in athletes, thereby improving their performance and recovery. The recommended dose of omega-3s for athletes varies between 1 and 5 g per day of EPA and DHA combined, with a daily dose of 3 g appearing to be effective in reducing inflammation and oxidative stress in athletes [220]. Omega-3 supplementation can be used both in the pre-competition phase and in the recovery phase. A study showed that omega-3 supplementation for 6 weeks prior to an endurance event improved performance and reduced inflammation [221]. The duration of omega-3 supplementation varies between 4 and 12 weeks, depending on the study and the purpose of the supplementation, with 4 weeks of supplementation generally being sufficient to improve recovery and reduce oxidative stress in athletes [220].

In general, omega-3 supplementation may be an effective strategy to reduce oxidative stress in athletes and improve their performance and recovery, with a daily dose of 3 g of EPA and DHA combined for 4 weeks appearing to be sufficient to provide significant benefits. However, it is important to note that, as with any supplement, consulting a health professional before starting omega-3 supplementation is recommended.

### 11.8. Zinc

Zinc, an essential micronutrient, plays a crucial role in maintaining good health and athletic performance. It acts as an antioxidant by neutralizing free radicals and reducing oxidative stress. Zinc also regulates the immune system and protein synthesis, which can aid in muscle recovery after intense exercise. The recommended dose of zinc for athletes varies depending on the type of sport and the duration and intensity of exercise. Studies have shown that a daily dose of 15–30 mg of zinc can improve antioxidant status and reduce oxidative stress in athletes [222]. However, higher doses may not be beneficial and can have adverse health effects, such as immune system suppression and reduced nutrient absorption [223]. The timing of supplementation may also be important, with some studies suggesting that pre-exercise supplementation may be more effective in reducing oxidative stress compared to during or after exercise [222]. Additionally, the duration of treatment may play a role, as zinc supplementation for at least 4–6 weeks has been shown to improve antioxidant status in athletes [224].

In conclusion, zinc supplementation may have beneficial effects in reducing oxidative stress and improving exercise performance under certain conditions [222]. Effective doses vary among studies, but generally, daily doses of 15–30 mg of zinc have been used. Pre-exercise supplementation appears to be more effective, although the optimal duration of treatment is still being researched, with varying ranges of 4–12 weeks. It is important to note that excessive zinc supplementation can be toxic, and it is recommended not to exceed the recommended daily intake of 40 mg. Therefore, finding the balance between an effective dose and a toxic dose should be considered when supplementing zinc in athletes.

### 11.9. Glutathione

Glutathione, an endogenous antioxidant produced by the body and found in various cells, including skeletal muscle cells, has been studied for its effects on oxidative stress and exercise performance. Interestingly, the effective dose of glutathione appears to depend on the method of administration. In one study, oral supplementation with 450 mg of glutathione per day for 3 weeks improved antioxidant status [225]. On the other hand, another study showed that intravenous administration of a single acute dose of 3000 mg/day of glutathione improved total antioxidant capacity [226]. Additionally, a study using a daily dose of 1000 mg for three weeks found a reduction in serum levels of oxidative stress markers [227]. However, more studies are needed to determine the optimal dose of glutathione to improve athletic performance and reduce oxidative stress.

The timing of glutathione supplementation may also be important, but evidence suggests that it can be taken acutely or chronically (up to 4–6 weeks) [225]. Further studies are needed to determine the optimal duration of glutathione supplementation. Moreover, more research is required to investigate the different types of glutathione and their absorption and bioavailability in the body.

In general, while glutathione supplementation has shown some beneficial effects in reducing oxidative stress and improving athletic performance, the results are inconsistent, and more research is needed to determine the effective dose, the timing of supplementation, and the duration of treatment. It is important to continue studying glutathione supplementation to better understand its potential benefits for athletes. Similarly, N-acetylcysteine (NAC) has been used as a precursor of glutathione to potentially enhance performance in elite sports, but concerns about its side effects with high doses have been raised [228]. The scientific literature suggests that NAC supplementation in the range of 1.2–20 g/day, whether taken acutely or over several weeks, can produce performance effects that vary from beneficial to trivial or even detrimental, and the true performance effect of NAC remains unclear. Additionally, the extent to which NAC may cause side effects is not fully understood, although doses greater than 5 g may be associated with more side effects than doses less than 2 g. Therefore, further research on NAC supplementation and its effects on athletic performance is needed before definitive conclusions can be made [228].

In general terms, it has been observed that supplementation with combinations of antioxidants can be effective in improving antioxidant protection and reducing oxidative stress in athletes and the general population, potentially leading to improved athletic performance [229,230]. However, it is important to note that the effects may vary depending on the type of sport and the level of training, and it is recommended to consult a healthcare professional before starting any type of supplementation [231,232,233].

## 12. Practical Applications

This review provides valuable insights for the practical application of sports performance and understanding the actions of supplements that attenuate and regulate the effects of reactive oxygen species (ROS). Specifically, it helps to comprehend performance from a holistic perspective, including microbiology and the relationship between antioxidant capacity and the microbiota, as well as the potential benefits of an antioxidant-rich diet and antioxidant supplementation for sports performance. As such, this review may lead to the implementation of new working methods and dietary protocols in sports teams aiming to improve performance.

## 13. Conclusions

There is growing evidence suggesting that ROS production during physical activity greatly influences sports performance. This review concludes that ROS plays a critical role in the processes of training adaptation induced by resistance training, through a reduction in inflammatory mediators and oxidative stress, as well as appropriate molecular signaling. Additionally, it has been established that micronutrients play an important role in counteracting free radicals, such as reactive oxygen species, which cause oxidative stress, and the effects of antioxidants on recovery, sports performance, and strategies for using antioxidant supplements, such as vitamin C, vitamin E, resveratrol, coenzyme Q10, selenium, and curcumin, to enhance physical and mental well-being.

## Figures and Tables

**Table 1:** Summary of the main antioxidants together with their physiological function, their effect on sports performance, and the scientific evidence.

Antioxidant	Physiological Function	Effect on Sports Performance	Scientific Evidence
Vitamin C	Helps protect cells from oxidative damage and improves iron absorption	Possible improvement in aerobic capacity	Small
Vitamin E	Protects cell membranes from oxidative damage	Possible improvement in aerobic capacity	Small
Quercetin	Anti-inflammatory, antidiabetic, antioxidant, anti-infective, and cardioprotective effects	Improvement in exercise capacity (VO_2_ max and endurance exercise performance)	Small
Resveratrol	Neuroprotective and cardioprotective effects	May improve endurance capacity and skeletal muscle strength	Small
Beetroot juice (nitric oxide)	Hemodynamic (vasodilator) and metabolic functions (promotes oxygen transfer in the muscle)	Improves cardiorespiratory performance at the anaerobic threshold and VO_2_ max intensities	Medium
Coenzyme Q10	Cellular energy production, antioxidant protection, regulation of the immune system, and improvement of cardiovascular and cognitive function	Possible improvement in aerobic capacity	Small
Spirulina	Strengthening the immune system, lowering cholesterol, and regulating blood sugar	Possible improvement in oxygen uptake and exercise tolerance at submaximal intensities	Small
Polyphenols	Enhances the production of vasodilating factors and blood flow	Enhancement of vascular function, muscle perfusion, and oxygen extraction during exercise	Small

**Table 2:** Antioxidant content in food.

Vitamin E (mg/100 g)		Vitamin C (mg/100 g)		Vitamin A (mcg/100 g)		Zinc (mg/100 g)		Selenium (mcg/100 g)	
Sunflower oil	55	Kiwi	500	Animal vetches	5800	Cooked oysters	37	Brazil nuts	1917
Maize oil	31	Guava	480	Sorrel	2100	Wheat germ	12.2	Pork kidneys	311
Wheat germ	30	Red pepper	204	Carrots	2000	Hemp seeds	9.9	Lamb	218
Hazelnuts	26	Red currant	200	Spinach (cooked)	1000	Roast beef	8.5	Eggs	192
Almonds	25	Parsley	150	Parsley	1160	Almonds	5	Oysters	154
Coconut	17	Persimmon	130	Butter	970	Peanuts	4.8	Veal	141
Corn germ	16	Brussels sprouts	100	Sweet potatoes	670	Cooked veal liver	4.5	Turkey	144
Soybean oil	14	Lemon	80	Soybean oil	583	Cooked turkey	4.5	Mustard seed	208
Soya bean sprouts	13	Cauliflower	70	Fresh and frozen tuna and bonito	450	Cooked veal	4.4	Sunflower seeds	78.2
Olive oil	12	Spinach	60	Cheese	240	Cooked chicken liver	4.3	Garlic	14.2
Margarine	10	Strawberry	60	Eggs	220	Cooked crab	4.3		
Peanuts	9	Orange	50	Other vegetables	130	Cooked lamb	4		

## Data Availability

Not applicable.
